# Supplementary material for: Prevalence of anxiety, depression, stress and insomnia among healthcare professionals during COVID-19 in Africa: umbrella review of existing meta-analyses
Source: PeerJ. 2024 Oct 30;12:e18108. doi: 10.7717/peerj.18108 (PMC11531257; doi:10.7717/peerj.18108)
Supplement: Supplemental Information 2 [file peerj-12-18108-s002.docx]

**The rationale for conducting the Umbrella review**

The ongoing COVID-19 pandemic has affected healthcare systems everywhere, particularly in Africa, with challenges that have never been seen before. In Africa healthcare professionals were with various mental health problems during COVID-19. However, very little was done on the extensive evidence regarding mental disorders. The purpose of this umbrella review was to provide comprehensive data on the prevalence of anxiety, depression, stress, and insomnia among healthcare professionals during the COVID-19 pandemic in Africa.

**The contribution that it makes to knowledge in light of previously published related reports, including other Umbrella reviews.**

To our knowledge, this is the first umbrella review to examine the pooled prevalence of depression, anxiety, stress and insomnia on healthcare professionals during COVID-19 in Africa. Healthcare professionals in Africa were affected by a variety of mental health problems during COVID-19 pandemic. The findings provide evidence that the prevalence prevalence was much higher in the Africa compared to those reported elsewhere. This research provide thorough data on the impact of the pandemic on the mental health of healthcare professionals in Africa. This study findings highlight the need to develop appropriate public health interventions to address healthcare professionals mental health needs in Africa during COVID-19 and related crisis..
