# Supplementary material for: Prevalence of anxiety, depression, stress and insomnia among healthcare professionals during COVID-19 in Africa: umbrella review of existing meta-analyses
Source: PeerJ. 2024 Oct 30;12:e18108. doi: 10.7717/peerj.18108 (PMC11531257; doi:10.7717/peerj.18108)

**Supplementary figure 1**: Graphical presentation of assessment of study overlap in reviews using Corrected Covered Area (CCA) method


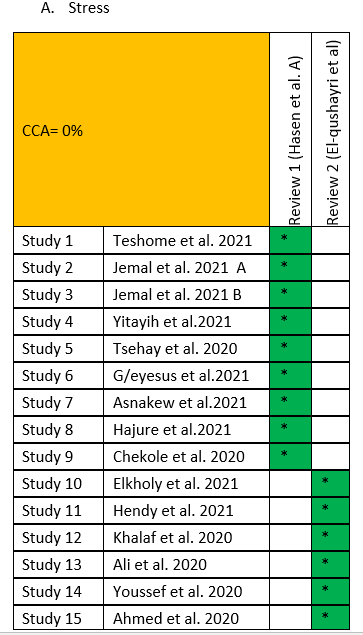

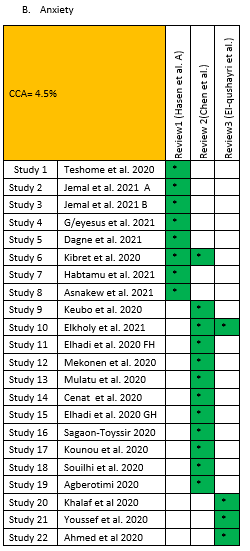


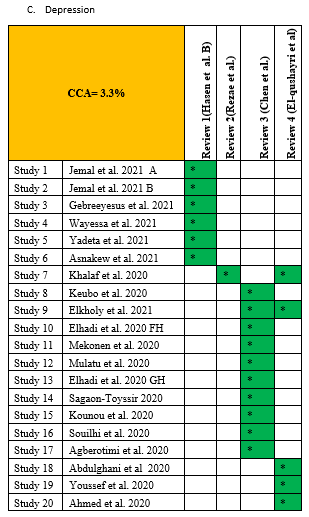

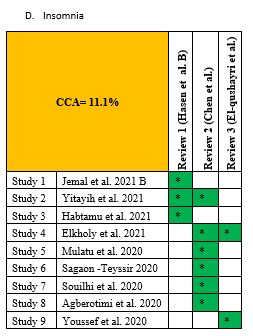

Supplement: Figure S3 — Supplementary figure 1 [file peerj-12-18108-s003.docx]
